# Incretin-responsive human pancreatic adipose tissue organoids: A functional model for fatty pancreas research

**DOI:** 10.1016/j.molmet.2024.102067

**Published:** 2024-11-14

**Authors:** E. Lorza-Gil, O.D. Strauss, E. Ziegler, K. Kansy, M.-T. Katschke, G. Rahimi, D. Neuscheler, L. Sandforth, A. Sandforth, G. Sancar, B. Kaufmann, D. Hartmann, S. Singer, A.L. Mihaljevic, R. Jumpertz-von Schwartzenberg, J. Sbierski-Kind, T.D. Müller, A.L. Birkenfeld, F. Gerst

**Affiliations:** 1 German Center for Diabetes Research (DZD e.V.), Germany; 2 Institute for Diabetes Research and Metabolic Diseases of the Helmholtz Center Munich at University of Tübingen, Tübingen, Germany; 3 Department of Internal Medicine IV, Division of Endocrinology, Diabetology and Nephrology, University Hospital Tübingen, Tübingen, Germany; 4 M3 Research Center, University Hospital Tübingen, Tübingen, Germany; 5 Institute for Diabetes and Obesity, Helmholtz Diabetes Center, Helmholtz Munich, Neuherberg, Germany; 6 Department of General, Visceral and Transplant Surgery, University Hospital Tübingen, Tübingen, Germany; 7 Department of Pathology and Neuropathology, University Hospital Tübingen, Tübingen, Germany; 8 Walther-Straub Institute of Pharmacology and Toxicology, Ludwig-Maximilians University Munich, Munich, Germany

**Keywords:** Pancreatic adipose tissue, Organoids, Adipogenesis, Incretins, Inflammation

## Abstract

**Objective:**

Infiltration of adipocytes into the pancreatic parenchyma has been linked to impaired insulin secretion in individuals with increased genetic risk of T2D and prediabetic conditions. However, the study of this ectopic fat depot has been limited by the lack of suitable *in vitro* models.

**Methods:**

Here, we developed a novel 3D model of functionally mature human pancreatic adipose tissue organoids by aggregating human pancreatic adipose tissue-derived stromal vascular fraction (SVF) cells into organoids and differentiating them over 19 days.

**Results:**

These organoids carry biological properties of the *in situ* pancreatic fat, presenting levels of adipogenic markers comparable to native pancreatic adipocytes and improved lipolytic and anti-lipolytic response compared to conventional 2D cultures. The organoids harbour a small population of immune cells, mimicking *in vivo* adipose environment. Furthermore, they express GIPR, allowing investigation of incretin effects in pancreatic fat. In accordance, GIP and the dual GLP1R/GIPR agonist tirzepatide stimulate lipolysis but had distinct effects on the expression of proinflammatory cytokines.

**Conclusions:**

This novel adipose organoid model is a valuable tool to study the metabolic impact of incretin signalling in pancreatic adipose tissue, revealing potential therapeutic targets of incretins beyond islets. The donor-specific metabolic memory of these organoids enables examination of the pancreatic fat-islet crosstalk in a donor-related metabolic context.

## Highlights

• Pancreatic adipose organoids retain the phenotype of native adipocytes.
• GIPR agonism stimulates lipolysis in human pancreatic adipocytes.
• The organoids harbor immune cells and secrete proinflammatory chemokines.
• Tirzepatide and GIP have distinct effects on pancreatic adipose tissue inflammation.

## 1 Introduction

Excessive lipid accumulation in adipose tissue triggers hypertrophy and stress of adipocytes, leading to infiltration of proinflammatory immune cells, fibrosis and adipocyte cell death, collectively referred to as adipose tissue dysfunction [1,2]. As consequence, adipocytes capacity to store lipids is impaired and fat is ectopically accumulated in organs such as muscle, liver and pancreas, a condition that promotes organ dysfunction and insulin resistance, contributing to the pathogenesis of type 2 diabetes (T2D) [3,4].

Although fat accumulation in human pancreas was described decades ago [5,6], it has for long remained an underexplored facet of ectopic fat distribution [7,8]. Pancreatic fat has been associated with improved insulin secretion in normoglycaemic subjects, but with impaired insulin secretion in patients at increased risk of T2D [[9], [10], [11]]. Furthermore, T2D diabetes remission, i.e. recovery of beta cell function was accompanied by reduction of pancreatic fat [12]. These clinical observations point to the controversial role of pancreatic fat in insulin secretion, and emphasize the need for experimental evidence demonstrating plausible lipolysis derived fatty acids-/secretome-mediated effects of pancreatic adipocytes in islets. To date, detailed studies on the mechanistic interactions between pancreatic adipocytes and insulin secretion remain sparse, as reliable *in vitro* models replicating the unique properties of these cells have been lacking [[13], [14], [15]].

Increased visceral adiposity and ectopic fat are clinical manifestations in patients with obesity and T2D [16,17]. During the last decade, the incretin hormone GLP-1 (glucagon like peptide-1) and GIP (gastrointestinal peptide) receptors have received great attention as effective pharmacological targets for counteracting T2D and obesity [[18], [19], [20]]. Thus, pharmacological tailoring of visceral and ectopic fat by mono/dual agonists of GLP1R and GIPR, such as semaglutide and tirzepatide, respectively, seems to preserve beta cell function, in addition to the well-known augmentation of insulin secretion. It is, however, unclear whether and to what extent pancreatic adipose tissue is involved in this effect. Intriguingly, there is no convincing evidence for functionally relevant expression of the GLP1R in adipose tissue [[21], [22], [23]], while GIPR is predominantly localized in non-adipocytes, particularly in immune cells [21,24,25]. GIPR signalling modulates adipogenesis, lipid storage and immune cell activity but the underlying mechanisms are not thoroughly elucidated [26]. Specifically, data on effects of incretins in specific adipose tissue depots such as pancreatic adipose tissue is not available, since no functional *in vitro* models of human pancreatic adipocytes have been developed so far. Previous attempts to characterize pancreatic adipocytes *in vitro* yielded unsatisfactory results, because human pancreatic preadipocytes have low adipogenic capacity and the *in vitro* differentiated cells (2D cell culture) failed to mimic the adipogenic and lipolytic phenotype of native pancreatic adipocytes [27,28]. In this work, we generated pancreatic adipose tissue organoids that display physiological characteristics of native pancreatic adipocytes. To achieve this goal, we used 3D cell culture method and stromal vascular fraction (SVF) cells derived from human pancreatic fat biopsies. Our data show that this newly developed *in vitro* model of pancreatic adipose tissue can serve as robust tool for investigating (patho)physiological traits of this adipose tissue depot, including the effects of GLP-1, GIP, and dual GLP-1/GIP receptor agonists, which currently are the best in class pharmacological agents to treat T2D and obesity. Moreover, our model also serves to investigate the role of pancreatic adipose tissue in beta cell function and T2D pathophysiology in a donor-specific metabolic context.

## 2 Material and methods

### 2.1 Patient recruitment and human pancreatic resections

Human pancreatic resections were obtained from 13 donors (HbA1c ≤ 5.7%; fasting glucose <5.7 mM) undergoing pancreatic surgery at the University Hospital Tuebingen. All patients gave their informed written consent. Donors characteristics are provided in Suppl. Table S1. Experiments performed in this study were approved by the Ethics Committee of the Medical Faculty of the Eberhard Karls University and the University Hospital Tübingen in accordance to the Declaration of Helsinki (697/2011B01).

### 2.2 Isolation of human pancreatic adipocytes and stromal vascular fraction (SVF) cells, and *in vitro* expansion and adipogenic differentiation of SVF-derived preadipocytes

**Figure 1:**
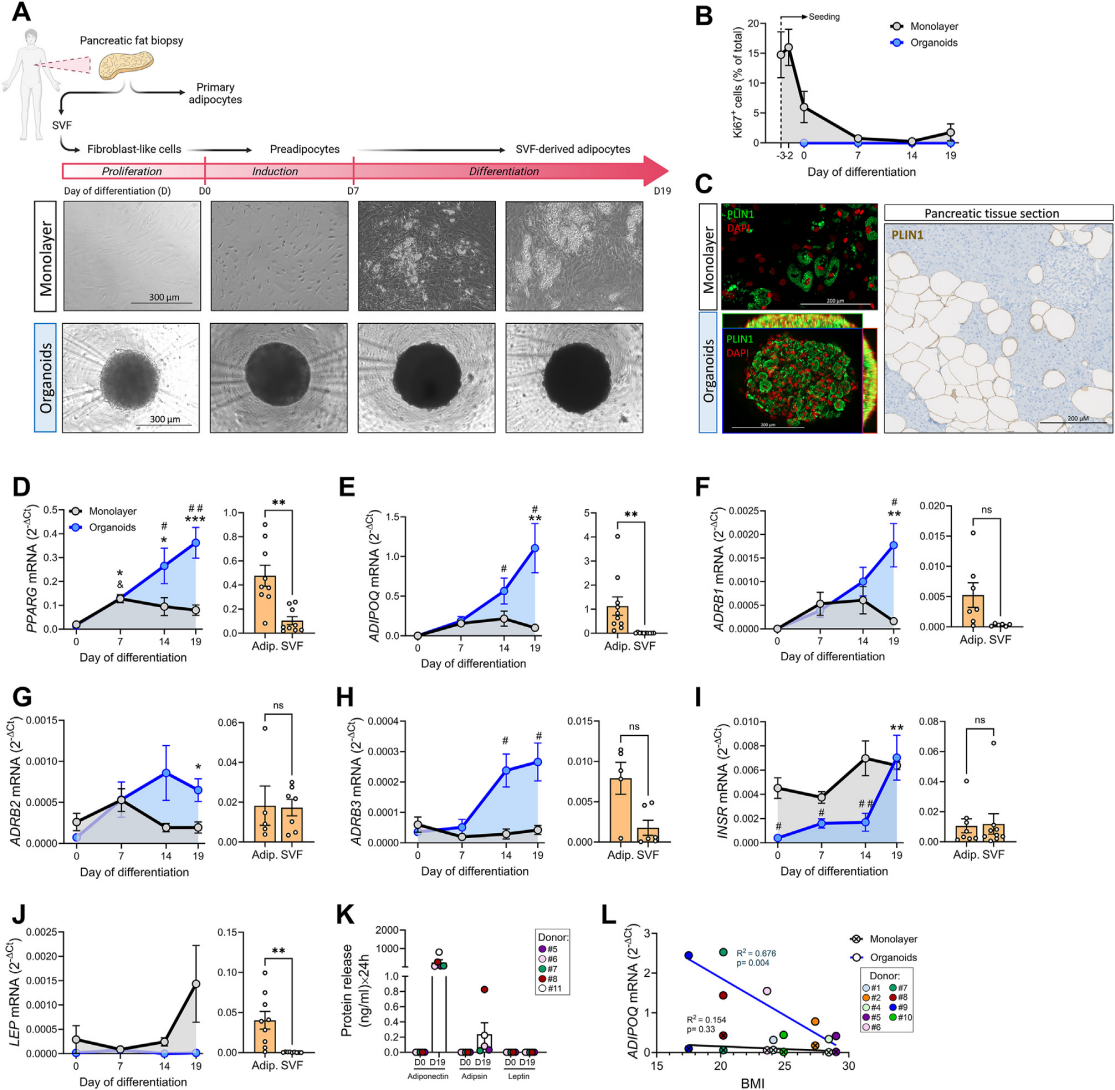
**Pancreatic adipose tissue organoids display improved adipogenesis. (A)** Schematic representation of *in vitro* generation of functionally mature pancreatic adipocytes: human pancreatic fat tissue is digested with collagenase to isolate primary mature adipocytes and SVF cells. SVF cells are expanded, seeded onto conventional adherent plates (2D; monolayer cell culture) or ultra-low-attachment plates (3D; spheroid cell culture) and subjected to adipogenic induction (D0, day 0) for 7 days, followed by a 12-day differentiation period. Representative brightfield images of 2D and 3D cells at D0, D7, D14 and D19. Scale bar 300 μm. **(B)** Percentage or proliferative (Ki67^+^cells) during 2D and 3D differentiation. Results are expressed as mean ± SEM for *n* = 4–5. No proliferation was detected in organoids. **(C)** Representative microscopy images of *in vitro* differentiated mature adipocytes (monolayer and organoids; confocal fluorescent image) and of *in situ* mature adipocytes (human pancreatic tissue section; brightfield chromogenic image) immunostained for the lipid droplet membrane protein perilipin-1 (PLIN1). Nuclei are stained in red. Scale bar 200 μm. **(D–J)** Relative mRNA levels (RT-PCR) of selected genes in the course of (left panels) cell differentiation as monolayer (grey lines) or organoids (blue lines) and (right panels; orange bars) in the primary adipocytes and SVF cells. Following genes were analyzed: **(D)***PPARG*, (E) *ADIPOQ* (adiponectin), **(F)***ADRB1* (beta 1 adrenergic receptor), **(G)***ADRB2* (beta 2 adrenergic receptor), **(H)***ADRB3* (beta 3 adrenergic receptor), **(I)***INSR* (insulin receptor) and **(J)***LEP* (leptin). *RPS13* was used as housekeeping. Results are presented as mean ± SEM for *n* = 8–10 donors. Statistical analysis was done using two-way ANOVA; &*p* < 0.05 vs D0 monolayer; ∗*p* < 0.05 vs D0 organoid; #*p* < 0.05 monolayer vs organoids. **(K)** Secreted adipokines by organoid cell cultures at D0 and D19 of differentiation. Results are expressed as mean ± SEM for *n* = 4 donors. **(L)** Pearson correlation of *ADIPOQ* mRNA levels at D19 with the donor’s BMI for monolayer and organoids of *n* = 9 independent donors.

Peri-pancreatic fat tissue was minced and digested with collagenase (Typ CLS I; 250 U/ml in a buffer containing: 1.5% BSA, 100 mM HEPES, 120 mM NaCl, 50 mM KCL, 1 mM CaCl2, 5 mM glucose, pH 7.4) for max. 30 min at 37 °C. Digested tissue was filtered (100 μm mesh) and centrifuged (5 min; 800 rpm) to separate mature buoyant adipocytes from SVF. SVF cells were cultured in AlphaMEM/Ham’s F12 (1:1) supplemented with 20% FBS, 1% chicken embryo extract, 1% penicillin/streptomycin, amphotericin (0.5 mg/ml) and expanded to 80% confluence. Thereafter, cells were seeded as 2D (monolayer) or 3D (spheroid) cultures. For monolayer culture, cells were cultured in standard adherent 24-well plates (4 × 10^4^ cells/well) and grown to 100% confluence. For spheroid culture, 5000 cells/well were seeded in ultra-low attachment (ULA) 96-well plates (Figure 1A). We refer to pancreatic adipose tissue-derived SVF cells cultured using the spheroid system as pancreatic adipose tissue organoids. Adipogenesis was initiated as previously described [28]. Briefly, SVF-derived organoids or monolayers were cultured for 7d in induction media (IM) consisting of DMEM/Ham’s mixture F12 (1:1) supplemented with (μmol/l): 17 panthothenate, 1 biotin, 0.025 apotransferrin, 1 insulin, 500 IBMX, 1 dexamethasone, 5 troglitazone, 50 indomethacin; 5% (v/v) FBS and 1% penicillin/streptomycin. After induction period, cells were cultured for additional 12d in differentiation media (DM) consisting of IM deprived of IBMX, dexamethasone and indomethacin. During adipogenic differentiation in the presence of GIPR/GLP1R agonists, the organoids were cultured in standard culture media supplemented with GIP (20 nM) or tirzepatide (10 nM) starting from day 7 of differentiation. Culture media was refreshed every 2nd day.

### 2.3 Immunohistochemistry

Organoids and monolayer cells were fixed with 4% formalin-PBS, permeabilized with 0.2% Triton X-100-PBS, blocked in 10% FBS-PBS, and incubated overnight with primary antibodies against PLIN1 (1:100), ATGL (1:800), HSL (1:200), CD68 (1:200), collagen IV (1:50) and collagen VI (1:200) and Ki67 (1:200), followed by 2 h-incubation with anti-rabbit/anti-mouse Alexa-Fluor488/546-IgG (1:2000). Nuclei were stained with DAPI (1 μg/ml) and triglycerides with BODIPY (5 μmol/l) or Oil red O (0.3%). For generation of organoid cross-sections, formalin-fixed spheroids were embedded in Histogel, dehydrated (70%-85%-95%–100% ethanol, 100% xylene), embedded in paraffin and cut in 4 μm thick sections. The sections were deparaffinised, rehydrated with alcohol series (100% xylene, 100% iso-propanol, 96%-85%-70% ethanol, H2O) and immunostained as described above. Confocal fluorescent imaging (2D and Z-stacks) was performed with an ApoTome System (Zeiss; 20x). Ki67^+^ proliferative cells were counted and expressed as % of total number of cells.

### 2.4 Lipolysis assay

Organoids and monolayer cells at differentiation day 19 (D19) were starved for 3 h in DMEM/Ham’s nutrient mixture F12 (1:1) supplemented with 0.5% free fatty acids (FFA)-free BSA. Subsequently, cells (5 organoids/100 μl; monolayers: 300 μl/well) were incubated for additional 3 h in Krebs–Ringer–HEPES (KRH) buffer (containing in mmol/L): 135 NaCl, 4.8 KCl, 2.6 CaCl2, 1.17 KH2PO4, 1.18 MgSO4, 5 NaHCO3, 10 HEPES, 5 glucose, and 0.5% (wt/vol) FFA-free BSA; pH 7.4 in the presence of forskolin (5 μM), isoproterenol (1 μM), human insulin (10 nM), tirzepatide (10 nM) or GIP (100 nM). Released FFA and glycerol were quantified with commercial fluorometric assays. For protein extraction, cells were lysed in RIPA buffer supplemented with protease inhibitors, and protein concentrations were measured by Bradford assay. Secreted FFA and glycerol were normalized against respective protein amounts.

### 2.5 Secretome analysis

Differentiated organoids (D19) were preincubated for 4 h with FBS-free DMEM/F12 supplemented with 0.5% FFA-free BSA, followed by additional culture (24 h; 10 organoids/100 μl) in the presence of GIP, tirzepatide and isoproterenol. 24 h-culture media was collected and cells were lysed for RNA extraction. Secreted cytokines and adipokines were quantified using Bio-Plex Pro Human Cytokine Assay and Pro Human Diabetes Assay.

### 2.6 Lipopolysacharide (LPS) treatment

Organoids (10 organoids/100 μl) and monolayer cells (100 μl/well; 96-well plate) at D19 were preincubated for 1 h with Cli095 (5 μM) in DMEM/F12 (1:1) supplemented with 0.5% FFA-free BSA. Following preincubation, the cells were treated with LPS (100 ng/ml) ± Cli095 for 24 h. Thereafter, cells were lysed for RNA extraction and the media was collected for secretome quantification.

### 2.7 RNA isolation and semi-quantitative real-time (RTqPCR)

Mature primary adipocytes (freshly isolated from pancreatic biopsies), SVF cells and *in vitro* differentiated organoids (12–20/sample) and monolayer cells at D0, D7, D14 and D19 were lysed and RNA was isolated using Nucleospin RNA isolation kit. Following evaluation of integrity and concentration of RNA, cDNA of 0.05 μg RNA was synthesised using Transcriptor first strand cDNA synthesis kit. RT-qPCR was performed using PowerTrack SYBR Master Mix and the LightCycler 480 system (Roche Diagnostics) using primers provided in Suppl. Table S2.

### 2.8 Western blot

Cellular proteins generated in lipolysis experiments were extracted with RIPA, boiled with Laemmli buffer, separated on 10% SDS-PAGE, transferred onto nitrocellulose membranes, and blocked for 1 h with 5% non-fat dry milk in TBS-Tween20 (0.1%; vol/vol). Membranes were incubated overnight with primary antibodies against P-Ser473-PKB, PKB, P-Ser660-HSL, HSL and GAPDH (1:1000) followed by 1 h incubation with horseradish peroxidase-coupled secondary antibody (1:2000). Protein and phosphoprotein bands were quantified relative to housekeeping protein (GAPDH) using Image Lab 5.2.1 Software.

### 2.9 FACS analysis

Single cell suspensions were prepared from organoids at differentiation day 0 and 19. To isolate immune cells, 100 organoids were digested with liberase Tm (40 μg/ml; 10 min; 37 °C), passed through a 40 μm filter and centrifuged (2000 rpm; 2 min). Cell pellets were resuspended in ice-cold 2% FBS-PBS and incubated with conjugated-fluorescent extracellular antibodies against CD45, CD14 and CD206 (45 min; 4 °C) and co-stained with fixable viability dye eFluor 780 to discriminate dead cells. Cells were immunophenotyped on a LSRFortessa + HTS (High Throughput Sampler) flow cytometer (BD Biosciences). Cells of interest were FSC-A/SSC-A gated to exclude debris, followed by singlets and viable, live-CD45^+^ gating. Macrophages were identified as viable CD45^+^CD14^+^CD206^+^. Data were analyzed using FlowJo software (version 10.10) and compiled using GraphPad Prism (version 10.1.1).

### 2.10 Statistical analysis

Data are presented as means ± SEM. Each dot represents an individual donor/independent experiment. For statistical significance, one-way ANOVA with Dunnett’s test and two-way ANOVA with Sidák’s test were performed using GraphPad Prism.

Essential materials and reagents are listed in Suppl. Table S3.

## 3 Results

### 3.1 Pancreatic adipose tissue organoids display adipogenic and functional characteristics similar to mature adipocytes

To determine if our newly developed 3D pancreatic adipose tissue organoid model better reflects (patho)physiology than currently used *in vitro* systems, we generated pancreatic adipocytes organoids from pancreatic adipose SVF cells. The cells were seeded (3D spheroid or 2D monolayer culture) and allow to proliferate until reaching confluence (Figure 1A). Two days post–confluence (day 0, D0 of differentiation), both 3D and 2D cultures were subjected to a 19-day of differentiation protocol. In the monolayer cells, proliferation rate (Ki67^+^cells) was 16 ± 3.02% on confluence day (D-2), dropped to 6 ± 2.61% at D0 and further decreased to 1.75 ± 1.43% at the end of differentiation (D19). In contrast, no Ki67^+^ cells were detected in organoids at any of the analyzed time points (D0, D7, D19), suggesting complete suppression of proliferation (Figure 1B).

At D19, organoid cells displayed increased staining with the lipid droplet marker perilipin-1 (PLIN1; Figure 1C) and triglyceride dye oil red O (Suppl. Fig. 1A) compared to monolayer cells. Furthermore, the organoids generated own ECM, as indicated by positive staining for collagen IV and collagen IV (Suppl. Figs. 1C and D). This underscores the ability of the 3D system to more accurately mimic the *in vivo* adipose environment and physiological lipid droplet storage compared to the monolayer culture. Fully differentiated adipocytes were present throughout the organoid, without evidence of core necrosis (Suppl. Fig. 1B). However, in contrast to *in situ* pancreatic adipocytes (Figure 1C, human pancreatic section), *in vitro* differentiated adipocytes (monolayer and organoids) were multilocular (Figure 1C). Next, we evaluated expression level of key adipogenic markers of *in vitro* differentiated adipocytes and compared them to those of *in situ* adipocytes. During *in vitro* differentiation, the organoids significantly upregulated mRNA levels of *PPARG* (0.36 ± 0.06) and adiponectin (*ADIPOQ*) (1.11 ± 0.31), compared to the monolayer adipocytes (0.08 ± 0.022 and 0.1 ± 0.044, respectively), reaching levels similar to those of *in situ* adipocytes (0.47 ± 0.08 and 1.12 ± 0.38, respectively) (Figure 1D,E). Similarly, beta-adrenergic receptors (*ADRB1, ADRB2, ADRB3*) were significantly higher in organoids compared to monolayer adipocytes (Figure 1F–H). *INSR* mRNA levels, though significantly lower in organiods at the beginning of differentiation (D0-D14), increased to a level comparable to that of monolayer adipocytes at D19 (Figure 1I). Interestingly, in contrast to *in situ* adipocytes, leptin (*LEP* mRNA) expression was very low in monolayer adipocytes and almost undetectable in organoids (Figure 1J). To corroborate the gene expression results, we quantified the amount of adiponectin, adipsin and leptin secreted by D0 and D19 organoids. Consistent with gene expression, D19 differentiated organoids released high levels of adiponectin, low levels of adipsin and almost undetectable levels of leptin (Figure 1K). We found a significant negative correlation between donors BMIs and *ADIPOQ* mRNA levels of organoids (Figure 1L). These results suggest that pancreatic adipose organoids display enhanced adipogenesis, maintain a donor-related phenotype, and therefore better mimic the *in vivo* adipocyte phenotype than the monolayer culture.

### 3.2 Pancreatic adipose tissue organoids acquire beta-adrenergic- and insulin-responsive lipolysis

**Figure 2:**
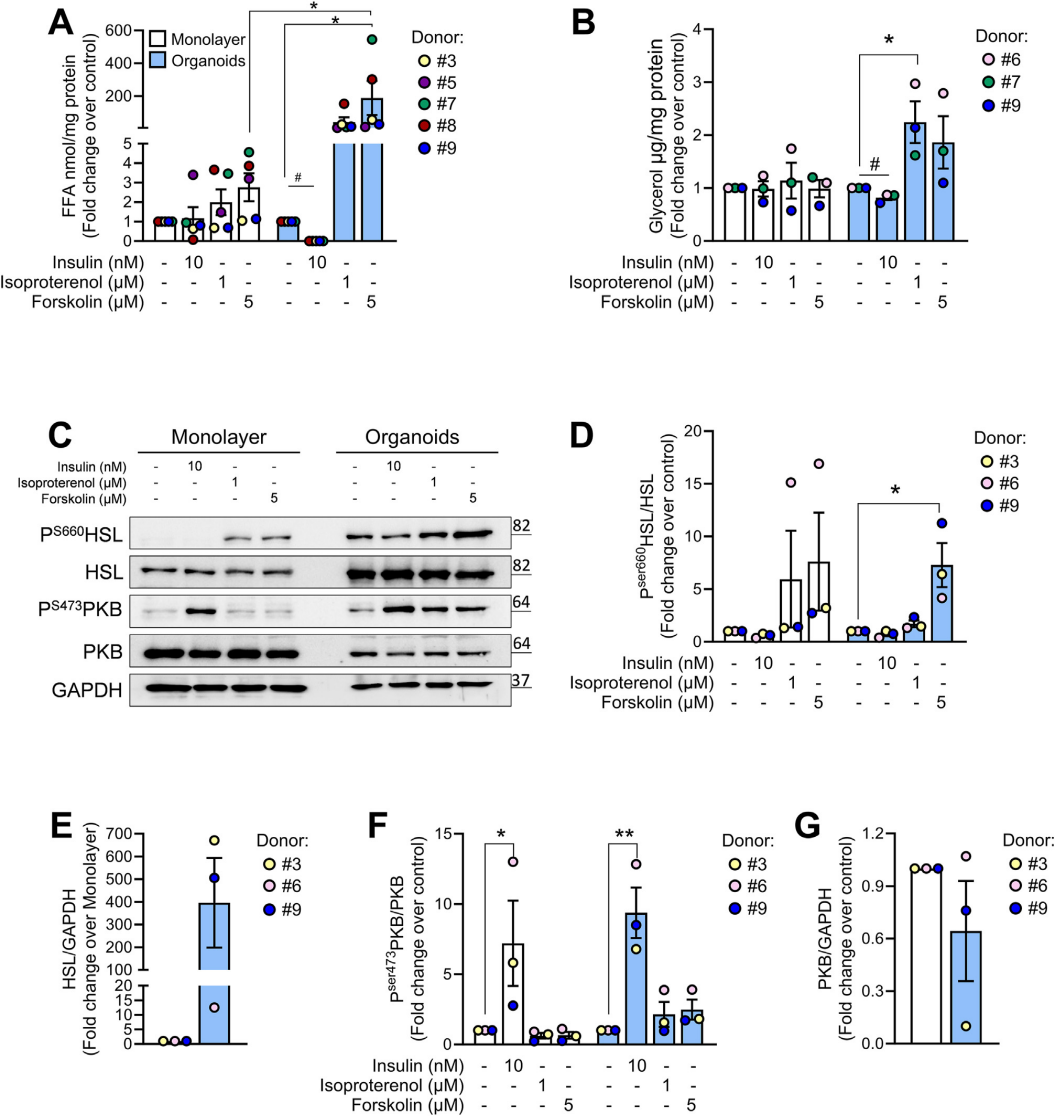
**Pancreatic adipose tissue organoids exhibit functional enhancements characteristic of mature adipocytes. (A–B)** Lipolytic performance of pancreatic adipocytes differentiated in monolayer (white bars) or as organoids (blue bars). The cells were incubated with insulin, isoproterenol and forskolin for 3 h, supernatant was collected and fatty acids and glycerol release was measured as described in the methods. Lipolysis was measured as release of **(A)** free fatty acids (FFA) and of **(B)** glycerol and presented as fold change over respective control. Results are presented as mean ± SEM for (A) *n* = 5 and (B) *n* = 3 independent donors. **(C–G)** Representative western blot **(C)** and relative quantifications **(D–G)** of P^Ser660^HSL, HSL, P^Ser473^PKB, PKB and GAPDH, in pancreatic adipocytes differentiated in monolayer (white bars) and organoid (blue bars) cultures. Results are expressed as FC (fold change) over respective control (Con) and presented as mean ± SEM of *n* = 3 donors. Statistical analysis was done using two-way ANOVA ∗*p* < 0.05, or student’s t-test #*p* < 0.05.

To evaluate whether improved differentiation of pancreatic adipose tissue organoids results in better functional response, we analyzed their lipolytic capacity. The major pro-lipolytic pathway is controlled by beta-adrenergic receptor (β-AR) signalling involving cAMP-dependent PKA activation and hormone-sensitive lipase (HSL)-mediated hydrolysis of triglycerides [[29], [30], [31], [32]]. Conversely, insulin suppresses lipolysis via reduction of cAMP levels through PKB-mediated activation of phosphodiesterase-3B [[33], [34], [35]]. Therefore, we assessed free fatty acids (FFA) and/or glycerol release as a readout of lipolysis in organoids and monolayer adipocytes (D19) in response to β-AR agonist isoproterenol (1 μM) and the adenylate cyclase activator forskolin (5 μM). Both drugs increased FFA release by 41- and 187-fold respectively in organoids, whereas monolayer adipocytes exhibited a modest increase, i.e. 2.0- and 2.8-fold, respectively (Figure 2A, Suppl. Fig. 2A). Similarly, an increase in glycerol release induced by isoproterenol and forskolin was observed in pancreatic organoids from all donors (Figure 2B, Suppl. Fig. 2B). Insulin (10 nM) effectively suppressed lipolysis in organoids only (Figure 2A,B). To address the molecular mechanisms underlying beta-adrenergic-induced, insulin-suppressed lipolysis, we assessed phosphorylation levels of HSL (serine 660) and PKB (serine 473). Isoproterenol and forskolin induced similar phosphorylation levels of HSL in organoids and monolayer adipocytes (Figure 2C,D; Suppl. Figs. 2C and D). However, the organoids expressed higher levels of HSL protein, which aligns with their superior differentiation potential (Figure 2C,E and Suppl. Figs. 2C, D, F). These findings suggest that the increased lipolytic ability of the organoids relies on increased expression of HSL, rather than on increased HSL activation. At a first glance, insulin-induced phosphorylation of PKB (FC over respective control) is similar in organoids and monolayers (Figure 2C,F, G and Suppl. Fig. 2C, D) implying similarly active insulin receptor. However, when PKB phosphorylation in monolayers was quantified relatively to PKB phosphorylation level in control-treated organoids (FC over organoid-control; Suppl. Fig. 2E), the organoids revealed higher levels of P-PKB, suggesting more effective insulin signaling.

### 3.3 Pancreatic adipose tissue organoids secrete proinflammatory chemokines

**Figure 3:**
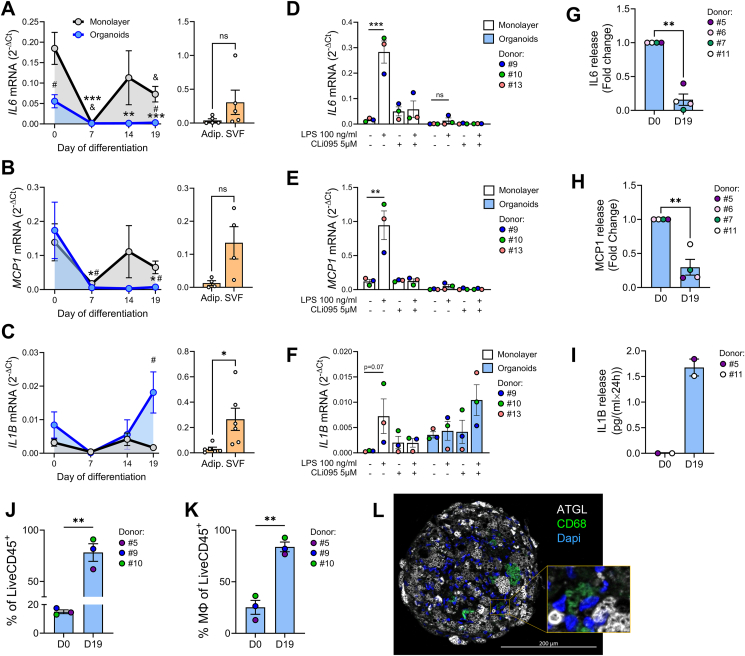
**Pancreatic adipose tissue organoids secrete proinflammatory chemokines. (A–C)** Relative mRNA levels assessed by RT-PCR of proinflammatory cytokines **(A)***IL-6*, **(B)***MCP-1* and **(C)***IL1B* during *in vitro* cell differentiation (left panels) as monolayer (grey lines) or as organoids (blue lines), and in the native adipocytes and SVF cells (orange bars, right panels). RPS13 was used as housekeeping. Results are presented as mean ± SEM for *n* = 4–9 donors. Statistical analysis was done using two-way ANOVA &*p* < 0.05 vs D0 monolayer; ∗*p* < 0.05 vs D0 organoids; *p* < 0.05 # monolayer vs organoid. **(D–F)** Relative mRNA levels (RT-PCR) of proinflammatory cytokines **(D)***IL-6*, **(E)***MCP-1* and **(F)***IL1B* in pancreatic adipocytes differentiated (D19) as monolayer (white bars) or organoids (blue bars). The cells were preincubated in the absence or presence of the TLR4 inhibitor, CLi095 (5 μM; 1 h) before LPS treatment (100 ng/ml; 24 h). Results are presented as mean ± SEM for *n* = 3 donors. **(G–I)** Secretion of **(G)** IL-6, **(H)** MCP-1 and **(I)** IL1B in organoids at D0 and D19. Results are presented as mean ± SEM for *n* = 3 donors. ∗*p* < 0.05 vs D0 organoids. **(J)** % of CD45^+^ leukocytes of viable organoid non-adipocyte cells and **(K)** % of macrophages in undifferentiated (D0) and differentiated (D19) organoids. Results are expressed as mean ± SEM for *n* = 3 donors. Student’s t-test; ∗∗p ≤ 0.01. **(L)** Representative fluorescent confocal image of a pancreatic adipocyte organoid section immunostained for ATGL (white), CD68 (green). Nuclei are stained in blue. Scale bar 200 μm.

As preadipocytes differentiate into mature adipocytes, their expression profile of inflammatory genes changes significantly [36]. Notably, native pancreatic adipocytes had lower mRNA levels of *IL-6*, *MCP-1* and *IL1B* compared to SVF (Figure 3A-C, right). The *in vitro* differentiated cells showed a significant downregulation of *IL-6* and *MCP-1* mRNA during induction period (D0-D7). However, during the later differentiation stage (D7-D19), their expression levels varied depending on the culture method. Thus, *IL-6* and *MCP-1* mRNA were upregulated in monolayers but remained low in organoids. In contrast, *IL1B* mRNA increased significantly in D19 organoids compared to the monolayers (Figure 3A–C, left). Of note, in D19 organoids, we found a positive correlation of *MCP-1* mRNA with donor’s NEFA (*p* = 0.04) and triglycerides (*p* = 0.05) levels, and of *IL1B* mRNA with triglycerides only (*p* = 0.01) (Suppl. Fig. 3).

After LPS exposure (24 h), *IL-6,* *MCP-1* and *IL1B* mRNA levels increased in monolayer adipocytes and TLR4 inhibition (CLI095; 5 μM) reduced LPS-induced expression of these cytokines. The organoids had a diminished inflammatory response to LPS exposure (Figure 3D,E). Consistent with the mRNA data, the protein levels of IL-6 and MCP-1 decreased in differentiated (D19 vs D0) organoids (Figure 3G,H). In contrast, IL1B protein level was elevated in differentiated organoids (D19 vs D0) aligning with the increased mRNA levels (Figure 3F,I).

Previous observations suggest that adipose tissue resident macrophages play a crucial role in cytokine production, and these SVF-derived macrophages might survive in spheroid culture [37,38]. To evaluate the immune cells in organoids, cell composition of non-differentiated (D0) and differentiated (D19) organoids was analyzed by flow cytometry (Suppl. Fig. 4). Interestingly, the % of CD45^+^ leukocytes of viable non-adipocyte cells increased 5.21-fold upon organoid differentiation, i.e. from 15 % at D0 to 78.17% at D19 (Figure 3J and Suppl. Fig. 4). In D0 organoids, 25.2% of CD45^+^ cells were CD14^+^CD206^+^ macrophages, while in D19 organoids the macrophage population increased to 83.8% of the CD45^+^ cells (Figure 3K). These observations were supported by identification of CD68-immunostained cells in D19 organoids (Figure 3L). However, we did not find other leukocyte subsets such as T-, B- or NK-cells (Suppl. Fig. 4).

### 3.4 GIPR agonism modulates lipolysis and inflammation in pancreatic adipocyte organoids

**Figure 4:**
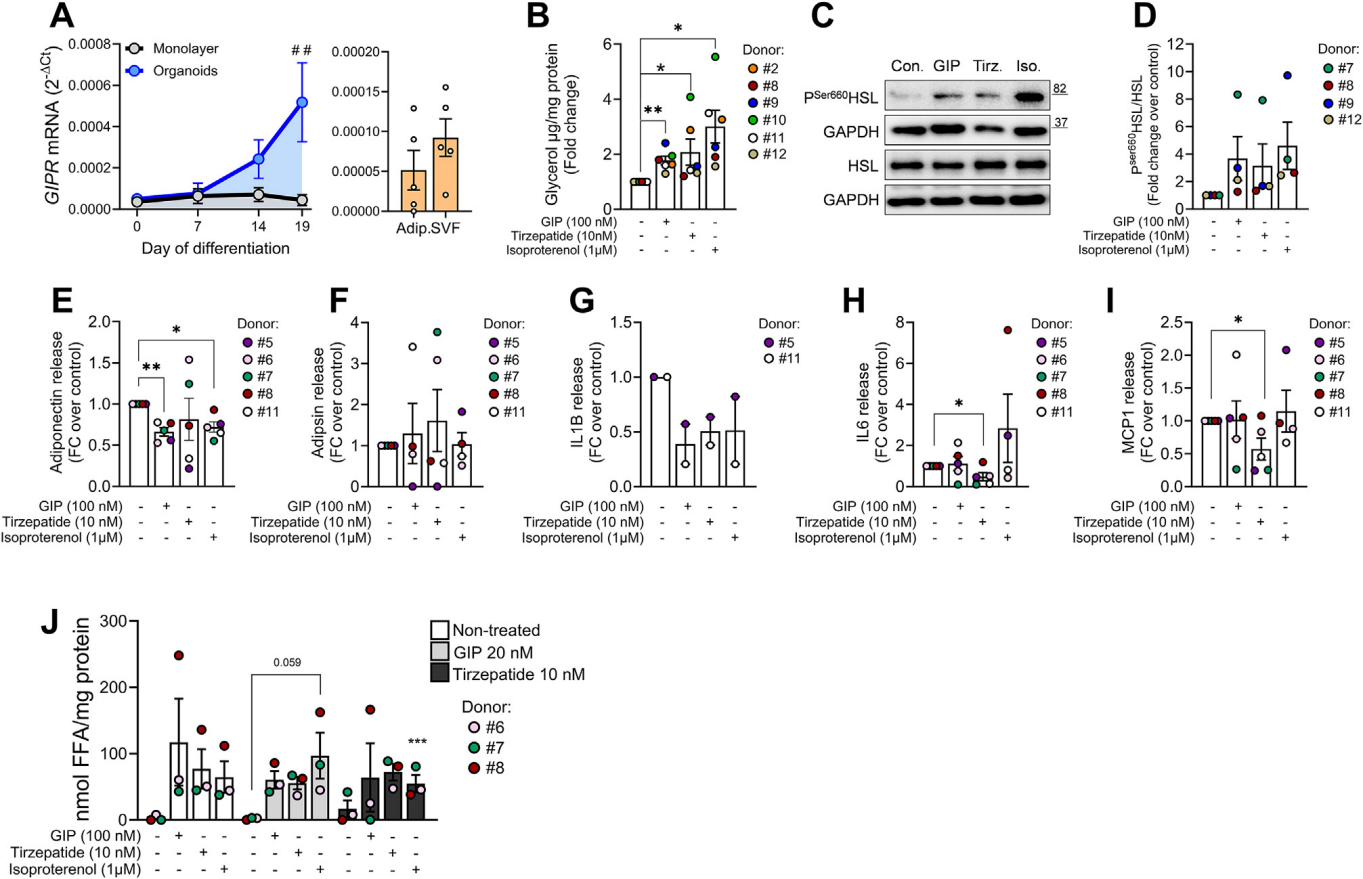
**GIPR agonism modulates lipolysis and inflammation in pancreatic adipose tissue organoids. (A)** Relative mRNA levels of *GIPR* (RT-PCR) during *in vitro* cell differentiation (left panel) as monolayer (gray line) or as organoid (blue line) culture and in the native adipocytes and SVF cells (right panel). Results are expressed as mean ± SEM for *n* = 4 independent experiments. Statistical analysis was done using two-way ANOVA ##*p* < 0.01 monolayer vs organoid. **(B)** Lipolytic performance of pancreatic adipocyte organoids quantified as glycerol release as described in the methods. **(C, D)** Representative immunoblots of **(C)** P^Ser660^HSL and HSL and **(D)** relative quantification P^Ser660^HSL in the organoids used for the lipolytic assays presented in **(B)**. Results are expressed as mean ± SEM of *n* = 4 independent experiments. **(E–I)** Secretome analysis in organoids at D19 after 24 h treatment with test substances as indicated. Supernatant was collected and protein levels of **(E)** adiponectin, **(F)** adipsin, **(G)** IL1B, **(H)** IL-6 and **(I)** MCP-1 were measured as described in the methods. **(J)** Lipolytic performance of pancreatic adipocyte organoids quantified as released free fatty acids (FFA) as described in the methods. Organoids were treated during differentiation (from D7 to D19) with GIP (20 nM) or tirzepatide (10 nM). At D19, cells were starved (3 h) and exposed to GIP (100 nM), tirzepatide (10 nM) or isoproterenol (1 μM) for another 3 h. Supernatant was collected and released FFA were measured and normalized to respective protein amount. Results are presented as mean ± SEM for *n* = 3 donors. Statistical analysis was done using one-way ANOVA ∗*p* < 0.05.

White adipose tissue (WAT) comprises a heterogeneous cell population including adipocytes, preadipocytes, immune cells, endothelial cells and fibroblasts. Unlike GLP1R, GIPR is expressed in WAT [21,39], being predominantly localized in the non-adipocytes, notably in immune cells [24,25]. In line with this observation, pancreatic SVF cells had slightly higher levels of *GIPR* mRNA than native pancreatic adipocytes (Figure 4A, right). On the contrary, *GIPR* mRNA level increased in differentiated organoids (D0 vs D19), but not in monolayer differentiated adipocytes (Figure 4A, left). *GLP1R* mRNA was undetectable at any timepoint and culture method (data not shown). This advocates for a role of GIPR in adipocyte maturation and adipose tissue function highlighting the *in vivo*-like phenotype of pancreatic adipose organoids. To investigate the functional relevance of GIPR in differentiated organoids, cells were treated with GIP (100 nM) and tirzepatide (10 nM) for 3 h. Incretin concentrations were chosen based on published pharmacodynamic assays [40]. GIP and tirzepatide increased lipolysis, as evidenced by a 1.7- and 2-fold increase in glycerol release, respectively (Figure 4B), as well as phosphorylation of HSL (Figure 4C,D), indicating that GIPR stimulates lipolysis via cAMP/PKA-dependent pathway.

To assess the impact of incretins on adipocyte secretome, we exposed the organoids to GIP and tirzepatide for 24 h and quantified the secreted adipokines/chemokines (Figure 4E–I). GIP, similar to isoproterenol (30%), reduced adiponectin release by 34%, while tirzepatide showed donor-dependent effects (Figure 4E). Adipsin production remained unchanged (Figure 4F). IL-6 and MCP-1 levels were significantly reduced by tirzepatide only (Figure 4H–I). Both GIP and tirzepatide decreased IL1B release, though the organoids produced very low levels, with IL1B being below detection limit in 2 out of 4 donors. These results suggest distinct roles of tirzepatide and GIP in pancreatic adipose tissue inflammation.

Previous works have found opposing effects of GIP in adipose tissue, i.e. triglyceride storage in human subcutaneous adipose tissue and lipolysis in *in vitro* differentiated human subcutaneous adipocytes [41,42]. To investigate the long-term effects of GIP and tirzepatide on adipogenesis, we treated the organoids with GIP (20 nM) and tirzepatide (10 nM) during differentiation (D7 to D19; Figure 4J). Notably, GIP did not affect expression of genes related to adipogenesis, adipocyte function and cytokine production (Suppl. Figs. 5A, B, H-J), while tirzepatide reduced the mRNA levels of *INSR* and *ADIPOQ* (Suppl. Figs. 5B and C). As chronic incretin exposure did not change *GIPR* mRNA levels (Suppl. Fig. 5G), we explored whether chronic GIPR agonism leads to desensitization of GIPR. To assess this, we measured lipolysis in response to isoproterenol and acute incretin treatment. We found similar lipolytic activity in organoids differentiated in standard and incretin-supplemented media, indicating that activity and expression of GIPR were preserved (Figure 4J).

## 4 Discussion

In the present study, we established a new adipocyte organoid model using SVF cells isolated from human pancreatic adipose tissue resections of metabolically characterized normoglycemic donors. The successful differentiation of the pancreatic preadipocytes *in vitro* marks a significant advance in fatty pancreas research, and offers a physiological model for a better understanding of this specific and often neglected adipose tissue depot, including preclinical drug evaluation.

The difficulties associated with *in vitro* adipogenesis of human primary visceral preadipocytes have significantly hindered the progress in adipose tissue research, prior to the development of 3D cell culture [43]. In accordance, our previous attempts to differentiate human pancreatic primary preadipocytes using monolayer culture yielded challenging results [28]. In this work we successfully overcame some of these difficulties by using 3D cell culture. This approach led to consistently superior differentiation of pancreatic adipose tissue-derived SVF cells across all donors, whereas the variations in the degree of adipogenesis were donor-dependent. In line with this, adiponectin mRNA levels varied significantly among the donors (Figure 1E) but displayed a strong negative correlation with the donor’s BMI (Figure 1L), confirming a previously reported inverse correlation of obesity with adiponectin expression [44,45]. Importantly, this BMI-adiponectin correlation was not observed in the monolayer adipocytes, emphasizing the donor-related metabolic memory of the organoids, and reflecting pathophysiology.

Notably, leptin mRNA levels were very low in monolayer adipocytes and at detection limit in organoids (Figure 1J). Such low leptin expression may be attributed to our differentiation protocol which includes thiazolidinediones, a class of PPARG activators that negatively affect leptin production [46]. While mRNA levels of β-AR were higher in organoids than in monolayer adipocytes, insulin receptor mRNAs were similar in D19 organoids and monolayer (Figure 1). In accordance, isoproterenol augmented the lipolytic performance of organoids to a higher extent, but only the organoids were able to considerably reduce the basal lipolytic rate in response to insulin (Figure 2A,B), mirroring physiological regulation of *in vivo* lipid metabolism. The apparently stronger suppressive effect of insulin on FFA release (Figure 2A vs. B) may result from its stimulatory effect on FFA re-esterification and uptake. Insulin’s failure to effectively suppress lipolysis in monolayer adipocytes, despite effective phosphorylation of PKB (Figure 2C,F) may stem from either defective signalling downstream of PKB, or faulty insulin-induced recruitment of PKB-independent/PI3K-dependent signals necessary to repress lipolysis [47]. This impairment may be a consequence of incomplete differentiation [1]. Indeed, preadipocytes secrete a range of cytokines [48], which can induce insulin resistance and hamper their own adipogenic differentiation [[49], [50], [51]]. In monolayers we found elevated mRNA levels of *IL-6* and *MCP-1* (Figure 3A,B, D, E), with LPS-induced cytokine expression being TLR4-dependent (Figure 3E–F). In contrast, D19 organoids showed elevated IL1B expression that was TLR4-independent after LPS exposure (Figure 3C,F, I), indicating a distinct cellular source from that of MCP-1 and IL-6. We also found more macrophages in differentiated D19 organoids (Figure 3K), supporting the increased IL1B expression (Figure 3F,I). Of note, TLR4-independent pathways can promote IL1B production in adipose tissue macrophages via saturated FFA/ceramide-induced inflammasome activation [52]. The positive correlation between plasma triglyceride (TGs) and the mRNA levels of *IL1B* in D19 organoids (Suppl. Fig. 3I) implies that donor’s lipid metabolism affects pancreatic adipose inflammation.

GLP-1R and GIPR agonists are valuable enhancers of glucose-dependent insulin secretion (GSIS) [53,54]. Because GIP effectiveness on GSIS is lost in T2D patients [19], its potential as anti-diabetic/anti-obesity drug has been neglected for a while. This situation changed dramatically since the potent GIPR/GLP1R dual agonist tirzepatide has been developed [18]. We report here expression of GIPR in pancreatic adipose tissue organoids, and effects of GIPR agonism on lipolysis, adipokine secretion and immunomodulation (Figure 4), revealing a broader impact of incretin signalling within the pancreas. However, the specific cellular source of GIPR within adipose tissue remains unclear, complicating the efforts to determine how GIPR signalling regulates adipose metabolism [25,39,41,55,56]. A recent work conducted with human subcutaneous adipocytes and mouse adipose tissue [21] provided additional insights, showing that GIP and tirzepatide bind to adipocytes and promote lipolysis, a finding consistent with our results (Figure 4B–D). Here, we extend this finding for the first time to human pancreatic adipose tissue, a fat depot suspected to exert direct effects on insulin secretion via lipolysis-derived FFA signalling upon activation of lipolysis [7]. According to previous observations, GIPR activation lowers adiponectin and increases IL6 levels, respectively [41,57]. In our hands GIP reduced adiponectin, whereas the levels of IL-6 and MCP-1 remained unchanged in organoids. On the other hand, tirzepatide decreased IL-6 and MCP-1 levels significantly (Figure 4H,I). All in one, we identify pancreatic adipose tissue as potential target of incretin action. Nevertheless, additional work is needed to elucidate whether the discrepancies between GIP and tirzepatide originate in tirzepatide-activated GLP1R signalling or is adipose tissue depot specific.

In conclusion, this study introduces a novel human pancreatic adipose tissue organoid model, which successfully mirrors metabolic characteristics of *in vivo* pancreatic adipose tissue. This model extends the understanding of pancreatic adipose tissue biology, particularly in the context of diabetes and obesity, and is a promising tool for investigating (i) incretin effects in pancreatic adipocytes, and (ii) metabolic interaction of pancreatic adipocytes with the islets in a donor-related context, emphasizing their potential utility in personalized medicine approaches. These investigations are of immediate importance for our understanding of the (patho)physiology and pharmacotherapy of T2D and obesity.

## CRediT authorship contribution statement

**E. Lorza-Gil:** Writing – review & editing, Writing – original draft, Visualization, Validation, Supervision, Resources, Project administration, Methodology, Investigation, Formal analysis, Data curation, Conceptualization. **O. Strauss:** Methodology, Formal analysis. **E. Ziegler:** Methodology, Formal analysis. **K. Kansy:** Methodology, Formal analysis. **M.-T. Katschke:** Methodology, Formal analysis. **G. Rahimi:** Methodology. **D. Neuscheler:** Methodology. **L. Sandforth:** Resources. **A. Sandforth:** Resources. **G. Sancar:** Conceptualization. **B. Kaufmann:** Resources. **D. Hartmann:** Resources. **S. Singer:** Resources, Project administration. **A.L. Mihaljevic:** Resources, Project administration. **R. Jumpertz-von Schwartzenberg:** Resources, Project administration. **J. Sbierski-Kind:** Methodology, Formal analysis, Conceptualization. **T.D. Müller:** Funding acquisition. **A.L. Birkenfeld:** Supervision, Funding acquisition. **F. Gerst:** Writing – review & editing, Writing – original draft, Visualization, Validation, Supervision, Resources, Project administration, Methodology, Investigation, Formal analysis, Data curation, Conceptualization.

## Funding

This study was supported by a grant from the German Research Foundation (DFG) (RTG2816/1 to ALB and TDM) and grant (01GI0925) from the 10.13039/501100002347Federal Ministry of Education and Research to the German Center for Diabetes Research (DZD e.V.) RTG2816 awarded scholarships to OS and GR.

## Declaration of competing interest

The authors declare that they have no known competing financial interests or personal relationships that could have appeared to influence the work reported in this paper.

## Data Availability

Data will be made available on request.
