# Supplementary material for: Incretin-responsive human pancreatic adipose tissue organoids: A functional model for fatty pancreas research
Source: Mol Metab. 2024 Nov 14;91:102067. doi: 10.1016/j.molmet.2024.102067 (PMC11625218; doi:10.1016/j.molmet.2024.102067)
Supplement: Multimedia component 1 [file mmc1.rtf]

Incretin-responsive human pancreatic adipose tissue organoids: a functional model for fatty pancreas research
Lorza-Gil E, Strauss O, Ziegler E, Kansy K, Katschke M-T, Rahimi G_,_ Neuscheler D, Sandforth L, Sandforth A, Sancar G, Kaufmann B, Hartmann D, Singer S, Mihaljevic AL, Jumpertz-von Schwartzenberg R, Sbierski-Kind J, Müller TD, Birkenfeld AL, Gerst F


Supplementary Figure 1 Microscopy confocal images of differentiated organoids (D19) stained for (A) triglycerides with Oil red O (Z-stack; maximal projection), (B) the lipid droplet marker perilin-1 (PLIN1; green), (C) collagen VI (red) and perilipin 1(PLIN1; green) and (D) collagen IV (green) and triglyceride (Bodipy; red). Nuclei are stained in red (B). Scalebar: 200 µm. 


Supplementary Figure 2. (A-B) Lipolytic performance (raw data) of pancreatic adipocytes differentiated in monolayer (white bars) or as organoids (blue bars) and quantified as release of (A) free fatty acids (FFA) and of (B) glycerol as described in the methods. The cells were incubated with insulin, isoproterenol and forskolin for 3h, supernatant was collected and fatty acids and glycerol release was measured as described. Results are presented as mean ± SEM for (A) n=6 and (B) n=4 independent donors. In the insulin-treated organoids, the level of released FFA (A) was under detection limit in 5 out of 6 donors. (C-D) Western blots of P^Ser660^HSL, HSL, P^Ser473^PKB, PKB and GAPDH in pancreatic adipocytes from two donors (#3; #9) differentiated in monolayer and in organoid cultures. (E) Relative quantification of P^Ser473^PKB in monolayers and organoids treated as indicated. Results are expressed as FC (fold change) over control (Con)-treated organoids and presented as mean ± SEM for n=3 donors (F) Representative confocal images of human pancreatic adipocytes monolayer and spheroids cross-sections stained for HSL (red), triglycerides (Bodipy; green; monolayer) or the lipid droplet marker perililin-1 (PLIN1; green; spheroid). Scalebar: 200 µm.
A.	


                         B.                                                    


C.                            D.


E.                       F.


Supplementary Figure 3. Pearson correlations of the mRNA levels of proinflammatory chemokines with (A-C) donor's BMI, (D-F) plasma NEFAs and (G-I) plasma triglycerides concentrations at D19 of differentiation for adipocyte monolayers and organoids of n=9 independent donors.
Supplementary Figure 4. FACS analysis of adipocyte spheroid resident immune cells. (A) Representative contour plots with outliers for CD45^+^ leukocytes and CD45^+^CD14^+^CD206^+^ macrophages (ÌÖ) in organoids at undifferentiated (D0) and differentiated (D19) stages. (B) Representative contour plots showing no presence of additional leukocyte subsets in the organoids at D19. T cells are defined as LiveCD45+CD3+, B cells as LiveCD45+CD19+ and NK cells as LiveCD45+CD3-CD19-CD14+CD16+/-CD56dim/bright.


A.


B.


Supplementary Figure 5. Spheroid cell cultures were treated during cell differentiation (D7 to D19) with GIP (20 nM) or Tirzepatide (10 nM). At day 19, cells were harvested and lysed for RNA analysis. Relative mRNA levels (RT-PCR) of (A) PPARG; (B) ADIPOQ; (C) INSR; (D) ADRB1; (E) ADRB2, (F) ADRB3, (G) GIPR, (H) IL6, (I) MCP1 and (J) IL1B. RPS13 was used as a housekeeping gene. Results are presented as mean ± SEM of n=5 donors. Statistical analysis was done using one-way ANOVA * p<0.05.


Supplementary Table S1. Clinical characteristics of the pancreatic tissue donors
Donor identifier	Age	Sex	BMI	HbA1c (%)	Fasting glucose (mmol/l)	Insulin (pmol/l)	C peptide (pmol/l)	Triglycerids (mg/dl)	NEFA (µmol/l)	Total cholesterol (mg/dl)	
#1	59	m	24.11	5.1	5.6	80	460	348	1189	303	
#2	70	m	27.38	5.6	3.9	20	159	146	1048	269	
#3	69	m	20.90	5.2	4.9	42	549	92	561	128	
#4	83	f	28.44	5.5	4.7	13	270	208	1322	190	
#5	63	m	29.01	5.3	4.7	51	421	87	344	154	
#6	52	f	23.62	5.6	4.8	101	488	348	934	192	
#7	78	f	20.21	5.6	4.9	37	309	143	963	199	
#8	25	m	20.20	4.8	2.5	7	91	64	2000	162	
#9	81	f	17.50	4.9	5.7	35	249	361	2809	389	
#10	55	m	24.91	5.7	5.4	24	376	124	372	164	
#11	73	m	32.41	5.5	5.1	52	390	171	803	216	
#12	65	m	22.20	5.5	4.9	60	309	126	428	214	
#13	52	f	23.23	5.5	4.7	48	351	187	533	268	


Supplementary Table S2. RT-PCR primer sequences
Gene	Forward primer	Reverse primer	
ADIPOQ	GGTGAGAAGGGTGAGAAAGGA	TTTCACCGATGTCTCCCTTAG	
ADRB1	GTGGAAGATGGGTGGGTTAG	GAGCCACGATGATCGATTTTA	
ADRB2	CCATGTCCAGAACCTTAGCC	GATCTGCGGAGTCCATGC	
ADRB3	TCTACTCTGCGCTGGCTTTT	GGAAGAAAAGAGTGAGGATTAGGC	
GIPR	GGACAGGCCTGATCGCC	CCCCTTAGAGCCTGTCTCCG	
GLP1R	TGGATGTATAGCACAGCCGC	AGCTCAGAGAGTCCTGGTAGG	
GLUT4	CCTCTCCGTGGCCATCTTTT	GTCAGGCGCTTCAGACTCTT	
IL1B	CTGTCCTGCGTGTTGAAAGA	TTGGGTAATTTTTGGGATCTACA	
IL6	CAGGAGCCCAGCTATGAACT	GAAGGCAGCAGGCAACAC	
INSR	GCAACATCACCCACTACCTGGT	GAATGGTGGAGACCAGGTCCTC	
LEP	TTGTCACCAGGATCAATGACA	GTCCAAACCGGTGACTTTCT	
MCP1	AGTCTCTGCCGCCCTTCT	GTGACTGGGGCATTGATTG	
PLIN1	GTCAGCCGGACTTGAGGA	TTCTGGAAGCATTCGCAGGT	
PPARG	CGAGTCTGTGGGGATAAAGC	GGATCCGGCAGTTAAGATCA	


Supplementary Table S3. Key resources  
REAGENT	SOURCE	IDENTIFIER	
Antibodies (WB and IHC)	
Perilipin 1 (PLIN1)	Progene	651156	
Adipose triglyceride lipase (ATGL)	Cell Signalling Technology Danvers, MA	2138	
Hormone sensitive lipase (HSL)	Cell Signalling Technology	18381	
P-Ser660-HSL	Cell Signalling Technology	45804	
Collagen IV	Dako	M0785	
Collagen VI	abcam	ab182744	
Ki67	Invitrogen	MA5-14520	
P-Ser473-PKB	Cell Signalling Technology	9271	
PKB	Cell Signalling Technology	9272	
CD68	abcam	ab955	
GAPDH	Cell Signalling Technology	2118	
Alexa-Fluor488/546-IgG	GE Healthcare	A-11008/A-11035	
horseradish peroxidase-coupled secondary antibody	GE Healthcare	NA934	
Essential Drugs and Media	
Chicken extract embryo	Seralab	CE-650-DL	
Human Insulin	Lilly	HI0210	
DAPI	Life Technologies	H3570	
Bodipy	Invitrogen	D392	
Oil red O	Sigma Aldrich	O9755	
ProLong Glass Antifade	ThermoFisher	P36980	
Histogel 	Epredia	HG4000	
Tirzepatide	Biozol	MCE-HY-P1731 	
GIP	Merck	G2269	
LPS	Sigma-Aldrich	L4391	
CLI095	Invivogen	tlrl-cli95-4	
Liberase Tm	Roche	5401127001	
Viability Dye eFluor 780	Invitrogen	65-0865-14	
UltraComp eBeads	Invitrogen	01-2222-42	
AlphaMEM	Sigma	M4526	
Ham´s F12	Gibco	31765-027	
DMEM/Ham's mixture F12	Gibco	31330-038	
FBS	Sigma	S0615	
Commercial Assays 	
FFA Assay Kit	Sigma-Aldrich	MAK126	
Glycerol Kit	Sigma-Aldrich	MAK117-1KT    	
protease inhibitors cocktail	Merck	11836170001	
Bradford assay	abcam	Ab119216	
Nucleospin RNA isolation kit	Macherey Nagel	740902.250	
RNA integrity kit	Agilent Technologies	5067-1513	
Transcriptor first strand cDNA synthesis kit	Roche Diagnostics	0489703 0001	
PowerTrack SYBR Master Mix	Thermofisher	A46110	
Bio-Plex Pro Human Cytokine Assay	BioRad Laboratories		
Bio-Plex Pro Human Diabetes Assay	BioRad Laboratories		
Fluorophores-coupled cell surface antibodies (FACS)	
BV785-CD3	Biolegend	317329	
BV711-CD56	Biolegend	318335	
BV650-CD45	Biolegend	304043	
FITC-CD14	Life Technologies	MHCD14014	
PerCP/Cyanine5.5- CD206	Biolegend	321122	
PE/Cyanine7-CD11b	Biolegend	101216	
PE-CD16	Biolegend	360704	
APC-CD19	Biolegend	392504	
AlexaFlour700-CD11c	Biolegend	337220	
eFluor 780-FVD780	Life Technologies	65-0865-14	
Softwares	
Image Lab 5.2.1 Software	BioRad Laboratories		
FlowJo Software 10.10	BD Biosciences		
ZEN	Zeiss		
GraphPad Prism 10.1.1	GraphPad
